# Use of Animal and Animal Products for Rheumatoid Arthritis Treatment: An Explorative Study in Odisha, India

**DOI:** 10.3389/fmed.2019.00323

**Published:** 2020-01-14

**Authors:** Mousumi Samal, Krushna Chandra Sahoo, Sanghamitra Pati, Saumya Ranjan Tripathy, Manoj Kumar Parida, Bidyut Kumar Das

**Affiliations:** ^1^Regional Medical Research Centre, Indian Council of Medical Research, Bhubaneswar, India; ^2^Clinical Immunology and Rheumatology, Department of Medicine, Srirama Chandra Bhanja Medical College and Hospital, Cuttack, India

**Keywords:** rheumatoid arthritis, musculoskeletal disorders, traditional remedy, folk healing, indigenous treatment

## Abstract

Severe fatigue, pain, deformity, and disability, are the major concerns for rheumatoid arthritis (RA). The extreme pain experienced by the patients often force them to experiment with various indigenous substances including animals and animal products. However, there is little evidence on the use of animals or animal products as traditional medicine in RA. Hence, this study was aimed to explore the experience and perception of patients toward the use of animals and animal products for the treatment of RA. A qualitative, explorative study was conducted at the out-patient-department of Rheumatology of a tertiary care medical college and hospital at Cuttack, Odisha, India. Out of 113 patients with RA, 18 patients gave history of use of animal and/or animal products and were selected for in-depth interviews. The content analysis methods were used for data analysis. Four major categories emerged: (1) prevailing patterns of traditional treatment of RA using animals, (2) beliefs and values behind the traditional treatment of RA, (3) sources and traditional learning pathway of indigenous practices on RA, and (4) ethical aspects of the indigenous practice of using animals and/or animal products in the treatment of RA. This study revealed the practice of eating dead animals to get relief from RA. However, there was hardly any perceived positive outcome of the practice; which indicates the lack of awareness of rational, scientific, treatment, and prevalence of irrational and unethical practices for the treatment of RA. Hence, community awareness, social mobilization, and newer screening tools are necessary to improve the timely detection and prevention of irrational treatment practices among RA patients.

## Introduction

Rheumatoid arthritis (RA) is one of the most common inflammatory musculoskeletal disorders. It commonly affects joints of fingers, wrists, feet, and ankles. The chronic inflammation leads to joint destruction and permanent deformities. The major concern with RA is persistent pain and other adverse health consequences as a result of chronic systemic inflammation (1, 2). Hence, timely and rational treatment of RA is important (2). Unfortunately, there is always a delay in seeking treatment by patients from suitable and trained healthcare providers (3). The choice of untrained healthcare providers with inadequate knowledge about RA often leads to delay in referral to a rheumatologist (4).

In India, delay in treatment is multifactorial: inaccessibility to trained healthcare providers, delay in referral to a rheumatologist and patients' beliefs on traditional healing practices (3, 5). The cultural beliefs have a significant impact on the care-seeking behavior of patients suffering from musculoskeletal disorders. Most of these patients prefer complementary and alternative medicines (CAM) which are often self-designed and administered (6). However, the above issue is not given priority in primary healthcare agenda. As better primary healthcare development depends on needs of individuals as well as community as a whole (7); the health system should understand the contextual phenomenon behind the unmet needs of the community on translational medicine (8).

In a country like India, patients have a large choice of healthcare to choose: self-medication, religious and traditional healers, herbal treatment, informal healthcare providers, trained prescribers, and qualified rheumatologists (5, 9). Self-medication and care-seeking behavior related to religious and traditional healers often force patients to indulge in various indigenous practices. However, there is a paucity of evidence on various indigenous practices for the treatment of RA. Therefore, this study explored the patients' experience and perception toward the use of animals and/or animal products for the treatment of RA.

## Method

This study is nested within a larger study on care-seeking behavior for RA (10). A mixed-method explorative study was conducted in a tertiary care medical college and hospital (SCB Medical College) at Cuttack, Odisha, India. A total of 113 In-depth Interviews (IDIs) was conducted to explore the care-seeking behavior of patients with rheumatoid arthritis attending the out-patient department of Rheumatology from August 2016 to April 2017. Out of 113 patients, 18 patients who had used animals and/or animal products as traditional medicines for the treatment of RA were selectively chosen.

The IDIs were conducted in Odia language by the first author, who is fluent in the local language and has an educational background in nursing and public health. The co-investigators have different backgrounds—zoology, medicine, and public health. The first three authors have experience in conducting qualitative research and the last three authors have clinical experience in treatment of RA. All the IDIs were audio-recorded, transcribed verbatim and translated into English. The average duration of IDI was 20 min with a range of 15–45 min.

The data were analyzed using content analysis methods. The data were anonymized. During collection of data, the study team read transcripts and prepared initial notes and summaries of the interview. Line-by-line open-coding was conducted using MaxQDA (Berlin, Germany). During data analysis first and second authors independently analyzed the text, and finally all the codes and categories were triangulated by all authors. The categories that emerged from the study were identified by first identifying the codes and categorizing them based on similar characteristics. The standards for reporting qualitative research (SRQR) guideline was used for data collection and analysis.

This study was approved by the ethical review committees of the Regional Medical Research Center, Bhubaneswar, Odisha and SCB Medical College, Cuttack. The concerned authority of the hospital gave permission to conduct IDIs among the patients. After explaining the purpose of the study, written consent was obtained from all participants; thumb impressions were taken from illiterate participants.

## Result

The average age of the participants was 40 years (range: 35–50 years). Four patients had no formal education, seven patients had received primary education, five patients had received high school education and two patients had completed graduation. Two patients had private jobs, three were daily laborers, and rest were housewives. The average duration of rheumatoid arthritis was 7 years with a range of 1–19 years. All participants belonged to rural areas.

Four major categories emerged: (1) animals and/or animal products used as traditional medicine (Table 1), (2) beliefs and values behind the traditional treatment of RA, (3) sources and traditional learning pathway of indigenous practices on RA, and (4) ethical aspects of the indigenous practices on RA. The findings are presented under each major category with quotes from the participants at the end of each paragraph. Information by the authors in the quotations is clarified in square brackets and the locally used terminologies are written in Italics.

**Table 1:** Key findings on the use of animals and animal products for the treatment of rheumatoid arthritis in rural Odisha.

Category	Animals and animal product used as traditional medicine	Beliefs and values behinds indigenous treatment and its consequences	Sources and traditional learning pathway of indigenous practices on RA	Ethical aspects of the indigenous practice
Codes	Rotten mongoose, earthworm, goat legs, grey hornbill, crow pheasant, Indian jackal	The traditional belief, lack of modern treatment, watching others getting cured, family pressure, to reduce severe pain, unpleasant smell and taste, hesitation on the treatment process, high price, not cured, healers easily accessible, no prescription fees, side effect, severity increases	Traditional healers, community members, neighbors, relatives, family members, ancient tale, ancient customs, generation to generation, beliefs and faith	A paucity of the modern facility, lack of awareness, mobile health team, referral system, health system accountability

### Category 1: Animals and/or Animal Products Used as Traditional Medicine

The participants revealed that they had eaten meat of various dead animals and birds in order to get relief from rheumatoid arthritis. These animals included rotten mongoose, jackal, earthworms, goat legs and stomach, various types of birds such as Indian Grey Hornbill (*Kuchilakhai*), and crow pheasant (*Kumbhatua*).

Traditional healers kill animals and have unique preparations which are prescribed to patients seeking help. For example the mongoose is placed in a clay pot filled with curd and buried underground till it rots. The rotten meat of the mongoose is then provided to patients seeking traditional remedies in bottles with instructions to consume specific amounts with curd after having bath in the morning.

“*I was advised to eat rotten mongoose, I felt very bad; it was nasty practice, but what can I do. If by consuming nasty and unhealthy things (food/medicine), my pain will be a relieved, I can sleep happily” (Patient 8)*.

The earthworm is usually ground properly. The paste of earthworm is mixed with curd and spices like cardamom and clove. The mixture is provided to the patients without informing the composition. About five patients had consumed the legs and stomach of goats continuously for 4–6 months in order to get relief from pain. The Indian Grey Hornbill meat is prepared by mixing around 50 types of spices by the traditional healer and kept in a bottle for 4–5 days. The patient is advised to take the meat every day in a specified amount until the whole portion is finished. As per the patients, the cost of Indian Grey Hornbill meat was 5–8 thousand Indian rupees. Some of the patients were suggested to hunt birds for preparing the medicine. The bird crow pheasant is prepared by boiling it with cumin seeds without any oil. In Table 2, the detailed list of animals used for the treatment of RA is given.

**Table 2:** Details of animal and animal products used for the treatment of rheumatoid arthritis.

**Sl. No**.	**The scientific name of the animal (Common name) [Local name]**	**Number of patients used**	**Preparation procedure and treatment duration**	**Source of information**
1.	*Ocyceros birostris* (Indian Grey Hornbill) [Kochilakhai]	5	Boiled with 50 types of spices. Provided for 7 days	Family and community members
2.	*Centropus sinensis* (Crow pheasant) [Kumbhatua]	1	Boiling with cumin seeds. Provided for 8 days	Community members
3.	*Herpestes edwardsi* (Indian grey mongoose) [Neula]	2	Killed and rotten for 4–5 days; then mixed with curd. Provided for 15 days	Traditional healer
4.	*Capra aegagrus hircus* (Goat) [Chheli]	1	Boiled. Provided for 45 days	Family members
5.	*Canis aureus indicus* (Indian jackal) [Bilua]	1	Killed and rotten for 4–5 days; then mixed with curd. Provided for 10 days	Traditional healer
6.	*Lumbricus terrestris* (Earthworm) [Jia]	3	Grinding and mixing with curd and spices. Provided for 15 days	Traditional healer

### Category 2: Beliefs and Values Behind the Indigenous Treatment of Rheumatoid Arthritis

The reasons behind indigenous practices are traditional beliefs, lack of awareness and/or availability of modern treatment, rumors/anecdotes of other patients getting cured by the use of such practices, family pressure, and the irresistible urge to get relief from severe pain. Around five patients had traditional beliefs that the knee and joint pain will reduce in intensity after taking an Indian Grey Hornbill bird.

“*I heard from the villagers' that two to three patients were completely cured by the traditional healer. They advised me to seek care from them. I was having a problem; now I am cured” (Patient 7)*.

The reason for choosing indigenous practices varied from patient to patient. Some ate rotten mongoose and jackal meat because of family pressure. The patient who ate earthworm indicated her hesitation to take the potion. However, severe pain and suffering forced her to practice traditional medicine. She explained that in order to get relief from the disease, she was ready to do follow any advice.

“*I told that I will not drink earthworm paste because I hate them. I feel severe pain. So, whoever says anything, I do that…only to get relief from the sufferings” (Patient 6)*.

Although traditional healers are easily available and often do not charge any prescription fees, the major drawbacks of the indigenous animal preparations include unpleasant smell, bad taste, high price, and most importantly, lack of efficacy.

Lack of adherence to these traditional medicines was common. There were various reasons for discontinuing these traditional medicines. One person discontinued eating rotten mongoose because of its unpleasant smell and bad taste.

“*It smells so bad and tastes horrible. They will pour curd with rotten mongoose. It is difficult to eat” (Patient 8)*.

The patients who were taking earthworm paste discontinued the treatment on realizing of its ingredients. The idea of consuming earthworms was not agreeable. Most of the patients discontinued consumption of birds because of high price and lack of availability. Some of the patients claimed that they had doubts about the source of the meat since they did not experience the cure that they had been promised or heard from anecdotes. Some stopped treatment because they did not get the remarkable effect after eating the Indian Grey Hornbill.

Most patients expressed that indigenous treatment was ineffective or was aggravating the severity of their disease. One patient expressed reduction of pain after eating the flesh of Indian Grey Hornbill. However, the disease relapsed when she stopped treatment.

“*After eating rotten mongoose for fifteen days, the symptoms increased and there was no improvement in health. For satisfaction of my mind, I was eating that…it was a horrible job” (Patient 8)*.

### Category 3: Sources and Traditional Learning Pathway of Indigenous Practices on Rheumatoid Arthritis

The sources of empirical knowledge and information for indigenous practice on rheumatoid arthritis included traditional healers, community members, neighbors, relatives, and family members.

“*Everyone forced me – my sister-in-law, mother-in-law, and father-in-law; if I will eat rotten mongoose the pain will reduce, that's why I took rotten mongoose” (Patient 8)*.

The patients were also influenced to take recourse to indigenous treatment because of the pressure of family members who were motivated by hearsay. About 10 patients tried the indigenous practice due to family pressure who believed the anecdotes of other patients getting cured by following similar practices.

“*They told… in the village the other three people had this disease and they also ate* Indian Grey Hornbill *and got cured. So they told me and I ate” (Patient 7)*.

The basic traditional learning pathway that emerged for indigenous practice on rheumatoid arthritis were ancient tales and ancient customs that has passed from generation to generation, and beliefs and faith in traditional and religious healers. Most of the patients took herbal medicines because of beliefs and faith in traditional and religious healers. One patient who had consumed the flesh of the Indian Grey Hornbill revealed that he was made to believe an ancient tale of her grandfather getting cured of rheumatoid arthritis after consuming the flesh of the Indian Grey Hornbill.

“*My father told that Indian grey hornbill is best for joint pain. My grandfather was cured by eating that bird. So, I thought that I will be cured; but I did not get relief” (Patient 13)*.

### Category 4: Ethical Aspects of the Indigenous Practice on Rheumatoid Arthritis

Lack of modern treatment facilities in rural settings and lack of awareness among the patients were the main reasons for favoring indigenous practices for the treatment of rheumatoid arthritis in remote areas. They felt that lack of trained healthcare providers in remote rural areas left them with no option but to visit and believe whatever the traditional healer prescribed. Most patients also believed that there was no allopathic treatment for rheumatoid arthritis while traditional healers promised them a permanent cure. Few participants suggested that there is an enormous need for awareness campaigns to change the behavior of patients toward indigenous practice. However, most of the patients expressed the need for an adequate number of trained providers at primary health facilities who can diagnose RA early for facilitating early referral to a trained rheumatologist at the district hospitals. Some opined that mobile health teams in rural and remote areas could bring about a significant change in the early treatment of RA.

## Discussion

In this study, the patients used the flesh of Indian grey hornbill, crow pheasant birds, Indian grey mongoose, Indian jackal, and earthworm for the treatment of RA. The sources of empirical knowledge on the traditional belief of these indigenous practices were from traditional healers, community members, relatives, and family members. The main reasons behind indigenous practices were lack of modern healthcare facilities, promise of relief from severe pain or due to societal influence: anecdotes of others in the community getting relief or pressure from family members for accepting such practices. This study explored the consequence of indigenous practice, types of indigenous substances of animal origin used, empirical knowledge pathway, and health system accountability for indigenous treatment practices of RA.

The fatigue, pain, disability, and wretchedness are found as major concerns for RA (11). Hence, the patients seek various treatment options including CAMs. In most of the cases, the CAMs are various indigenous substances such as cat's claw and bee-venom acupuncture (12), hen's (*Gallus domesticus*) blood and camel's (*Camelus bactrianus*) blood as well as milk; jackal (*Canis aureus*) flesh; blood of Indian wild ass (*Equus hemionus*), spiny-tailed lizard (*Uromastix hardwickii*), hammerhead shark (*Zygaena blochii*) (13), bat's bone (Pteropus species), turtle (*Kachuga tutorial*), and earthworm (*Pheretima posthuma*) (14).

A study in South India described the use of various animals and/or animal products as medicine—for example, fat of garden lizard (*Alotes versicolor*) to reduce acute pain, bone marrow of goat (*Capra aegagrus*) to cure tonsillitis, flesh of crow pheasant (*Centropus sinensis*) for asthma and tonsillitis, roasted penis and meat of mongoose (*Mungos mungo*) to reduce body pain, impotence, and jaundice (15). Similarly, dried powder of earthworm has been used as an aphrodisiac, as well as for the cure of piles and jaundice (15). According to a review in India, jackal meat was used for the treatment of paralysis and arthritis, and jackal blood for treatment of asthma (16). Similarly, the cat (*Felis domesticus*)—the whole animal is stripped off and boiled and the resultant juice used for treatment of arthritis (16).

A previous study in India found blood from hen, camel, jackal, and Indian wild ass being massaged externally on aching parts of the body for healing arthritis while roasted flesh of jackal was being used to cure asthma and sciatica and the spiny-tailed lizard boiled in oil being applied externally to heal joint pain and rheumatic diseases. The fat of the hammerhead shark was also used externally to heal joint pain (13). According to a study in Rajasthan milk of the camel was used as a massage cream and turtle (*Kachuga tontoria*) carapace flesh ash of carapace was taken orally to heal rheumatic diseases (17). A study in Madhya Pradesh, India found fat of kite (*Milvus migrants*) and fat of nilgai (*Bos camelus*) was massaged on the joint in case of arthritis, rheumatic diseases, internal injuries, and paralysis (18). A similar result also found in a study in Nepal that fat of bat and hornbill (*Pteropus spp*.) was warmed and massaged externally on the affected sites of RA (14).

In the current study, the fine paste of earthworm was used to treat RA, similar to the study from Nepal that describes its use to treat chronic illnesses and children suffering from measles and typhoid (14). We found jackal and mongoose being used to treat RA after fermenting with curd. Similar fermentation of jackal flesh mixed with paddy grains and a small amount of yeast called “syalko raksi” was used for body massage to relieve aches due to gout and arthritis or even taken orally in Nepal (14). The same study also describes the consumption of cooked meat from jackals and sloths (*Melurus ursinus*) for the treatment of gout, and other rheumatic pain (14). Trichechida's fat was used to massage on the affected parts in the case of rheumatic diseases, snakebite, and sprains (19).

The major motivating factors of indigenous practice in our study were easy accessibility of healers, and no prescription fees; whereas the demotivating factors were high price and the poor response to treatment. A study in Canada found satisfaction related to support was linked with adaptive behavior, whereas disappointment was connected with maladaptive behavior (20). According to a study in the United Kingdom, delay in seeking medical advice among patients and delay in referral systems were the important factors for the gap in the onset of symptoms to treatment from trained providers (3). Kumar et al. in the United Kingdom described the influence of ethnicity for example “folk beliefs,” family and friends, and dietary manipulation as the major underlying reasons for treatment delay (21), which is similar to our findings.

Many patients in our study thought that it was difficult to recover from RA by allopathic treatment; however, indigenous treatment was a more promising therapeutic option for RA. A previous study in northern Australia emphasized the need for multidisciplinary holistic approaches focusing on culture-specific health determinants in order to understand the cultural influence on health-seeking behaviors. They suggested capacity building and engagement of public health practitioners' on indigenous health concepts (22). However, a study in Canada suggested that there is a necessity of public knowledge about early symptoms—the lay people should be aware of the signs, symptoms, and treatment of RA for ethical healthcare (9). According to our findings, the paucity of the modern facility in a rural setting and lack of awareness among the patients were key drivers for indigenous practice. The previous studies on RA suggested that lack of information about RA was a critical factor for the treatment delay (23, 24). Hence, RA patients need to be well-informed and educated on modern treatment, which is a core component of care (4); therapeutic patient education is one of the essential components of the health system (25). A qualitative study in eight European countries on six rheumatic conditions suggested that there is a need for disease-specific interventions through non-physician health professionals in case of musculoskeletal care (26). According to Cheraghi-Sohi et al., patient-centered models of care are crucial for rational treatment (27).

The key knowledge sources of indigenous treatment of RA in our study were ancient tales, customs, and belief or faith on religious or traditional healers. The cultural background has influenced the use of complementary and alternative management of RA (28, 29). In medical sociology, it is important to understand people's beliefs about etiology—their feelings, thought processes and content of the lay beliefs (30). However, it is difficult to verify the indigenous knowledge by scientific methods or adequately assess beliefs behind such treatment practices, as they are built on non-conventional criteria, methodologies, and philosophies based on traditional beliefs and customs (31). Misleading assumptions often result in divested community health. The laypeople are not experts and rarely skilled in matters of fact gathering. They can be incorrect about the causes and management of the illness (30). It was found that patients' beliefs about disease etiology were often faith-based; therefore, they trusted God to cure them rather than opt treatment from healthcare providers (32). Gathering information, speaking to others, and seeking alternative treatments (29) were common in the case of RA. The patients gathered knowledge from friends, family, local, untrained care-providers, and mostly from other patients (33). In our study, the patients ate rotten mongoose because of the “belief of an ancient tale” that people got cured of RA by eating rotten mongoose. Similarly, a study in the Dominican Republic showed that patients had learned about their disease and treatment practices mostly from friends and family (32).

## Conclusions

The care-seeking behavior of patients and their choice of health care modality, i.e., traditional indigenous measures vs. modern scientific methods plays an important role in the outcome of the disease. The treatment of chronic illnesses like rheumatoid arthritis is often influenced by patients' perspectives on such illnesses such as likely duration, symptomatology, severity, treatment urgency, and available facilities which dictate the choice of healthcare made by patients. Hence, to improve the quality of life among RA patients and to motivate them to move away from traditional indigenous practices and seek early modern allopathic assistance, it is important to increase community awareness, social mobilization, and mass screening. This will help to increase early detection of RA and prevent irrational and futile, indigenous treatment practices.

## Data Availability Statement

The datasets generated for this study are available on request to the corresponding author.

## Ethics Statement

The studies involving human participants were reviewed and approved by Regional Medical Research Center, Bhubaneswar, India. The patients/participants provided their written informed consent to participate in this study.

## Author Contributions

KS, SP, and BD designed the study. BD, ST, and MP contributed patients for the study. MS conducted, transcribed, and translated the interviews. KS and MS analyzed the data and prepared the manuscript. BD and ST contributed to the final revision of the manuscript. The manuscript has been read and approved by all the authors.

### Conflict of Interest

The authors declare that the research was conducted in the absence of any commercial or financial relationships that could be construed as a potential conflict of interest.
